# Effects of discontinuing different antiresorptive regimens on medication-related osteonecrosis of the jaw in patients undergoing dental procedures: a systematic review and network meta-analysis

**DOI:** 10.1530/EOR-2024-0133

**Published:** 2025-05-05

**Authors:** Kasidid Ruksakiet, Atthakorn Jarusriwanna, Wuttapon Sadaeng, Artit Laoruengthana, Thanyaporn Sang-Ngoen, Teerapon Dhippayom

**Affiliations:** ^1^ Department of Restorative Dentistry, Faculty of Dentistry, Naresuan University, Phitsanulok, Thailand; ^2^ Department of Orthopaedics, Faculty of Medicine, Naresuan University, Phitsanulok, Thailand; ^3^ Department of Oral Diagnosis, Faculty of Dentistry, Naresuan University, Phitsanulok, Thailand; ^4^ Department of Oral Biology, Faculty of Dentistry, Naresuan University, Phitsanulok, Thailand; ^5^ The Research Unit of Evidence Synthesis (TRUES), Faculty of Pharmaceutical Sciences, Naresuan University, Phitsanulok, Thailand; ^6^ Department of Pharmacotherapy, University of Utah College of Pharmacy, Salt Lake City, Utah, USA

**Keywords:** antiresorptives, bisphosphonates, denosumab, osteonecrosis of the jaw, osteoporosis, bone metastasis

## Abstract

**Purpose:**

- Controversy exists on whether a drug holiday is necessary for patients on antiresorptive medication for osteoporosis or bone metastasis and undergoing dental procedures to lower the risk of medication-related osteonecrosis of the jaw (MRONJ). This study evaluated the effects of discontinuing different antiresorptive regimens on MRONJ in these patients.

**Methods:**

- Publications from PubMed, EMBASE, Cochrane Library and EBSCO Open Dissertations were searched from inception to September 2023 following PRISMA guidelines, and the review was registered in PROSPERO. Eligibility criteria included clinical studies on the effects of continued and discontinued antiresorptive medications for osteoporosis or bone metastasis in patients undergoing dental procedures. The involved antiresorptive agents were oral bisphosphonates (BPs), intravenous (IV) BPs and denosumab (Dmab). Relative risk (RR) with 95% confidence interval (CI) was estimated using a random-effects model.

**Results:**

- Of the 2,590 records identified, six studies (*n* = 717) were included. Discontinued use of oral BPs had a lower MRONJ risk than discontinuation of IV BPs (RR = 0.05; 95% CI: 0.00–0.83) and continuation of IV BPs (RR = 0.03; 95% CI: 0.00–0.46). Continuing oral BPs also resulted in a lower MRONJ risk compared to both discontinuation and continuation of IV BPs, with RR = 0.04 (95% CI: 0.00–0.67) and RR = 0.03 (95% CI: 0.00–0.37), respectively. No significant difference was found between continuation and discontinuation of oral BPs, along with other comparisons.

**Conclusions:**

- A drug holiday may not be necessary before dental procedures for oral BPs. Temporary discontinuation of IV BPs or Dmab is also unlikely to reduce MRONJ risk compared to continued medication.

## Introduction

Antiresorptive agents such as bisphosphonates (BPs) and denosumab (Dmab) are primarily used to suppress the activity and function of osteoclasts. These medications are widely prescribed for patients with either osteoporosis or bone metastasis (1, 2). The indicated patients, drug selection, dosage and treatment duration of these drugs are recommended in several guidelines (3, 4, 5). In previous landmark studies, oral BPs (alendronate, risedronate, ibandronate) showed efficacy in strengthening bone and reducing the risk of vertebral fractures by 41–62% after 3 years of treatment (6, 7, 8, 9). Meanwhile, zoledronic acid, which is an intravenous (IV) BP, could prevent these vertebral fractures by up to 70% (10). Patients with Dmab treatment also had a lower risk of vertebral fractures by 68% after treatment for 3 years (11). For the treatment of bone metastasis, high-dose IV BPs and Dmab are preferred, with efficacy in reducing the risk of skeletal-related events (SREs) by 17–38% (12, 13). These antiresorptive drugs have been shown to be cost-effective, reducing morbidity and improving quality of life in patients with osteoporosis, as well as in those with bone metastasis (14, 15).

However, long-term treatment with these antiresorptive agents may provide potential adverse effects. Medication-related osteonecrosis of the jaw (MRONJ) is a rare but serious complication, with progressive bone destruction in the maxillofacial region that could devastate the jawbone (16). The pathophysiology of MRONJ, while not fully understood, appears to involve a complex interplay of various factors, including local infection or inflammation and alterations in bone turnover following exposure to antiresorptive agents (17). In general, the incidence of MRONJ in osteoporosis patients who receive oral BPs, IV BPs and Dmab varies from 1.04–69, 0–90 and 0–30.2 per 100,000 patient-years, respectively, while a higher incidence in metastasis patients is observed, with 0–12,222 per 100,000 patient-years in patients receiving IV BPs and 0–2,316 per 100,000 patient-years in patients prescribed Dmab (18). Significant factors associated with the frequency of MRONJ include patient age, type and duration of antiresorptive treatment, underlying condition (osteoporosis or bone metastasis), exposure to immunosuppressive therapies, oral hygiene and invasive dental procedures e.g., tooth extractions. A prior systematic review revealed that the occurrence of MRONJ following tooth extraction in cancer patients who were treated with high-dose IV BPs and Dmab varies between 11 and 50% (19).

This condition contributes to the controversial issue of whether temporary drug discontinuation or a drug holiday is required before dental procedures or not, as this protocol may affect bone health, especially a rebound risk of fragility fractures in osteoporosis patients or higher SREs and progression of bone metastasis in malignancy patients (20, 21, 22). Conversely, when patients receiving ongoing antiresorptive medications require tooth extraction or related dental procedures, clinicians indeed encounter challenging decisions regarding the potential risks associated with these interventions. In some cases, the risks of complications, including the development of MRONJ, may outweigh the benefits of proceeding with tooth extraction (23).

Although a recent meta-analysis demonstrated that a drug holiday would not minimize the risk of MRONJ in patients who were treated with antiresorptive agents and underwent tooth extractions (24), there is a lack of evidence comparing various types of antiresorptive agents and the route of administration in different drug holiday protocols. Understanding how different drug holiday strategies affect the risk of MRONJ among antiresorptive agents can inform clinical practice and improve patient outcomes. This study aimed to evaluate the effects of discontinuing different antiresorptive regimens on MRONJ in patients undergoing dental procedures.

## Materials and methods

This systematic review and network meta-analysis was conducted following the guidelines of the Preferred Reporting Items for Systematic Reviews and Meta-Analyses (PRISMA) 2020 statement (25). The review protocol was registered in the International Prospective Register of Systematic Reviews (PROSPERO; registration number: CRD42024523022).

### Search strategy

A comprehensive search was conducted on PubMed, EMBASE, Cochrane Library (Cochrane Central Register of Controlled Trials (CENTRAL)) and EBSCO Open Dissertations from inception to September 2023. No filters or search restrictions were applied. In addition, snowballing and citation tracking of studies included from the database search were performed on the Scopus database to ensure comprehensive coverage of relevant studies. The search queries were designed in accordance with each database platform using Boolean operators. Briefly, free-text and thesaurus were searched to cover the following three domains: i) antiresorptive agents; ii) dental procedures and iii) osteonecrosis. The comprehensive search strategies of all databases are presented in Appendix 1 (see section on Supplementary materials given at the end of the article).

### Study selection

The specific inclusion criteria for each component of the Population, Intervention, Comparison, and Outcome (PICO) were as follows: i) population: patients who received antiresorptive agents for the treatment of osteoporosis or bone metastasis and underwent dental procedures, ii) intervention: continuation of the antiresorptive agents, iii) comparison: discontinuation of the antiresorptive agents and iv) outcome: reported the occurrence of MRONJ after dental procedures assessed by clinical and radiographic findings. Studies included in this review were full-text clinical studies of any retrospective cohort, case-control or randomized controlled trials (RCTs). We applied no language limitations in the inclusion criteria.

All obtained records were pooled in the reference manager program (EndNote™ version 21, Clarivate™, UK). Duplicated records were then identified and removed. The remaining records were transferred into spreadsheets. Subsequently, two reviewers (KS and AJ) independently screened the titles and abstracts of these records. Eligible records were then retrieved for full text and independently evaluated by the same reviewers. Any disagreements or discrepancies between the reviewers were discussed to achieve consensus. If necessary, a third reviewer (WS) was consulted for a consensus on the eligibility of the records. The records that were excluded after full-text assessment were noted.

### Data extraction

Two reviewers (KS and AJ) independently extracted data from the included studies using predefined data collection spreadsheets. Any disagreements were resolved through discussion and, if needed, consultation with a third reviewer (WS) to reach a consensus. The extracted data included authors, year of publication, country of origin, study design, indication for antiresorptive treatment, characteristics of participants, details of the drug intervention and comparator, details of all reported outcomes, and financial support. If the necessary data were missing from the included studies, the authors of those studies were contacted directly via email for the information.

### Quality assessment

Two reviewers (KS and AJ) independently assessed the methodological quality of the included studies using the Risk of Bias in Non-Randomized Studies of Interventions (ROBINS-I) tool for non-RCTs (26) and the Risk of Bias in Randomized Trials (RoB 2) tool for RCTs (27). Any disagreements were resolved through discussion with a third reviewer (WS).

### Statistical analysis

The general characteristics of the included studies and effects on other outcomes e.g. undesirable effects and quality of life status, were reported narratively. The impacts on MRONJ across different comparisons were pooled and reported as relative risk (RR) with its correspondence 95% confidence interval (CI) using a random-effect model. Global network inconsistency was examined to identify any overall conflict between direct and indirect evidence. The relative efficacy of different modalities was ranked based on their probabilities of being the best treatment option using the surface under the cumulative ranking curve (SUCRA). The Cochran’s Q statistic and *I^2^* were used to determine statistical heterogeneity. In addition, clinical heterogeneity and network transitivity were assessed across the included studies based on potential effect modifiers, including patient age and percentage of female participants, duration of antiresorptive treatment and discontinuation, and follow-up period after dental procedures. All statistical analyses were performed by using STATA version 15 (StataCorp LP, USA). A significant level was set at a *P*-value less than 0.05.

## Results

### Study selection

**Figure 1:**
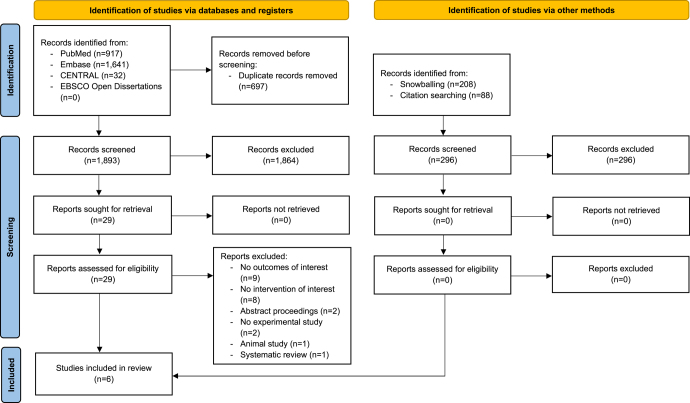
Flow diagram of selected articles according to the Preferred Reporting Items for Systematic Reviews and Meta-Analysis (PRISMA) guidelines.

Comprehensive database searches yielded 2,590 records. After removal of duplicated records, there were 1,893 records left for title and abstract screening. Twenty-nine records were eligible for full-text assessment, but 23 of them were excluded because they did not reach the inclusion criteria. Excluded studies are shown in Appendix 2 with their reasons. The remaining six articles were included in the final analysis (Fig. 1). Searching with other techniques yielded no additional articles.

### Study characteristics

The included studies consisted of four retrospective cohort studies (28, 29, 30, 31), one prospective cohort study (32) and one RCT (33). Of these, four studies prescribed antiresorptive agents for the treatment of bone metastasis (28, 29, 30, 33) and one study used them for osteoporosis treatment (32). The remaining study contained both conditions, but most of the patients were treated for osteoporosis (31). The total number of participants across the studies was 717, of which 595 (83%) patients were female. Patients in two studies received oral BPs (31, 32), whereas four studies involved IV BPs (28, 30, 31, 33), and Dmab was prescribed in two studies (29, 33). The mean age of patients ranged from 57.2 to 69.3 years, and the mean duration of antiresorptive treatment varied from 13.7 to 40.3 months (ranging from 2 to 96 months). The mean duration of discontinuation of antiresorptive agents before dental procedures ranged from 30 days to 39 months. The characteristics of the included studies are presented in Tables 1 and 2.

**Table 1:** Study and patient characteristics of the included studies.

Study	Country	Study design	Participants			Funding
			All	Females, *n* (%)	Mean age, years	
Bodem *et al.* (28)	Germany	RCS	61	42 (68.9)	65.7 ± 12.7	None
Cuozzo *et al.* (32)	Italy	PCS	45	43 (95.6)	67.5 ± 3.0	NR
Hasegawa *et al.* (29)	Japan	RCS	72	41 (56.9)	65.2 ± 11.8	NR
Kang *et al.* (31)	South Korea	RCS	465	420 (90.3)		Public funding
Males					63.7 ± 10.5	
Females					69.3 ± 8.8	
Karaca *et al.* (30)	Türkiye	RCS	51	37 (72.5)	57.2 ± 10.2	None
Ottesen *et al.* (33)	Denmark	RCT				Public funding
Zoledronate			10	5 (50)	67*	
Denosumab			13	7 (53.8)	69*	

RCS, retrospective cohort study; PCS, prospective cohort study; RCT, randomized controlled trial; NR, not reported.

* Median values.

**Table 2:** Indication, antiresorptive agent use, duration, results and follow-up details provided in the included studies.

Study	Indication	Antiresorptive agents		MRONJ after DP		Mean duration of ARST, months	Mean discontinuation period before DP, months	FUD after DP
		Name	Route	On drugs	Off drugs			
Bodem *et al.* (28)	Malignancy	Zoledronate, ibandronate, pamidronate	IV	7	1	40.3 ± 32.9	17.6 ± 15.9	12 weeks
Cuozzo *et al.* (32)	Osteoporosis	Alendronate, risedronate, ibandronate	PO	0	1	N/A	3	1 year
Hasegawa *et al.* (29)	Malignancy	Denosumab	SC	15	10			24 months
MRONJ						22.4 ± 13.5	66.0 ± 61.7 days	
No MRONJ						13.7 ± 15.3	58.9 ± 63.5 days	
Kang *et al.* (31)	Mainly osteoporosis	Alendronate, ibandronate	PO + IV	1 (IV); 0 (PO)	0	40.0 ± 35.6	39.0 ± 35.5	N/A
Karaca *et al.* (30)	Malignancy	Zoledronate, ibandronate	IV	2	1	24*	2*	At least 8 weeks
Ottesen *et al.* (33)	Malignancy	Zoledronate	IV	0	0	17.5*	1	6 months
		Denosumab	SC	0	4	9*		

* Median values.

ARST, antiresorptive treatment; DP, dental procedures; FUD, follow-up duration; MRONJ, medication-related osteonecrosis of the jaw; IV, intravenous; SC, subcutaneous; PO, per oral; N/A, not applicable.

### Quality assessment of the included studies

The quality of five non-RCTs based on ROBINS-I showed a ‘low’ overall risk of bias in one study (29) and a ‘moderate’ risk of bias in the other four studies (28, 30, 31, 32). This was attributed to the necessity for more explicit reporting of methodology and outcome assessments. Furthermore, the quality of the only included RCT (33) was justified as having ‘low’ overall risk of bias, since the risk of bias in all domains was low. The justification for all domains and the summary of ROBINS-I and RoB 2 are described in Appendix 3.

### Comparative risk of MRONJ

**Figure 2:**
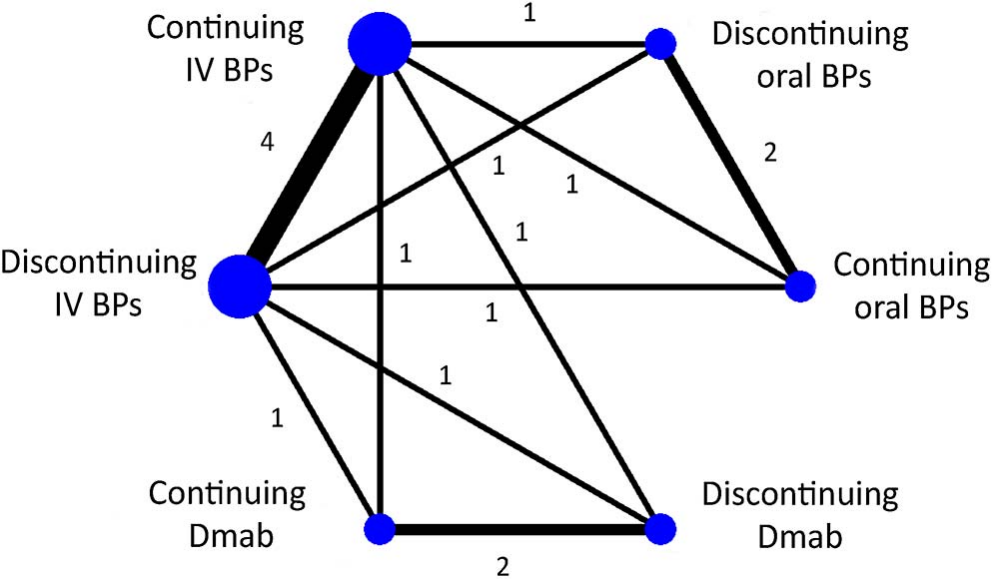
The network geometry of different antiresorptive administrations in the analysis for the MRONJ risk. The nodes in the network represent different antiresorptive treatments, and the edges (lines) between the nodes indicate direct comparisons made in the studies. The numbers on the edges represent the number of studies that directly compared the interventions. IV, intravenous; BPs, bisphosphonates; Dmab, denosumab.

A total of six interventions from six studies with 11 direct comparisons were included in the network meta-analysis (Fig. 2). According to the pooled estimates from different comparisons, patients continuing oral BPs significantly lowered the risk of MRONJ by 96% compared to those who discontinued IV BPs and by 97% compared to those who continued IV BPs (RR = 0.04; 95% CI: 0.00–0.67 for the discontinuing IV BPs, and RR = 0.03; 95% CI: 0.00–0.37 for continuing IV BPs). Conversely, the discontinuation of oral BPs also significantly decreased the risk of MRONJ by 95 and 97% when compared to discontinuation and continuation of IV BPs respectively (RR = 0.05; 95% CI: 0.00–0.83 for the discontinuing IV BPs and RR = 0.03; 95% CI: 0.00–0.46 for the continuing IV BPs). However, no statistical significance was observed between the discontinuation and continuation of oral BPs (RR = 0.81; 95% CI: 0.07–9.42), as well as the other comparative interventions including in-between groups of the discontinuation and continuation of IV BPs and Dmab (Table 3).

**Table 3:** The RR between different antiresorptive regimens.

**Continuing oral BPs**					
0.81 (0.07–9.42)	**Discontinuing oral BPs**				
0.04 (0.00–0.67)	0.05 (0.00–0.83)	**Discontinuing IV BPs**			
0.21 (0.01–6.56)	0.26 (0.01–8.09)	5.28 (0.72–38.76)	**Continuing Dmab**		
0.20 (0.01–6.27)	0.25 (0.01–7.74)	5.04 (0.69–37.05)	0.96 (0.51–1.80)	**Discontinuing Dmab**	
0.03 (0.00–0.37)	0.03 (0.00–0.46)	0.64 (0.19–2.18)	0.12 (0.01–1.22)	0.13 (0.01–1.27)	**Continuing IV BPs**

IV, intravenous; BPs, bisphosphonates; Dmab, denosumab; RR, relative risk.

The findings of significant effects from both continuing and discontinuing oral BPs were supported by the SUCRA ranking, which placed them as the top two interventions at 83.7 and 79.3% respectively (Appendix 4).

### Exploration for inconsistency and transitivity

There were no inconsistencies in the effect estimates between direct and indirect evidence within the network geometry (*P* = 0.488 for the inconsistency test). Transitivity assessment indicated similarity across the majority of different treatment comparisons. However, four out of 11 comparisons provided insufficient data to determine transitivity (Appendix 5).

### The other undesirable effects and quality of life status

Apart from the risk of MRONJ, only a study by Ottesen *et al.* (33) provided additional outcomes in bone metastasis patients who received either IV BPs or Dmab and had different drug holiday protocols. This study reported the patient’s quality of life, which was evaluated by EuroQol 5D-5L (EQ-5D-5L) questionnaire, in parallel with the occurrence of SREs and tumor progression. The median EQ-5D-5L scores of both medication groups (IV BPs and Dmab) demonstrated decreased health states in patients who had a drug holiday. For the SREs, 20% of patients in the continued Dmab group had a vertebral compression fracture and 12.5% of patients in the discontinued Dmab group had a spontaneous hip fracture. However, tumor progression was observed in 75% of patients who discontinued Dmab, whereas none of the continued Dmab patients and both different drug holiday protocols of IV BPs patients had tumor progression in the trial period.

## Discussion

Our study provides an extensive bibliographic review of the literature and demonstrates the comparative risk of MRONJ in patients who received different types and routes of antiresorptive agents and underwent dental procedures with varying drug holiday protocols. The strength of our study lies in the use of network meta-analysis, which considers both direct and indirect evidence to enhance the precision of our findings. This approach allows us to identify correlations, differentiate between administration routes and types of antiresorptive medications, and explore the comparative effects of different antiresorptive drug holiday regimens within the network geometry regarding the risk of MRONJ. Our study found that while drug holidays did not significantly alter the risk of MRONJ within the same type of antiresorptive regimen, patients who received oral BPs tended to have a lower risk of MRONJ following dental procedures compared to those receiving IV BPs.

Previous studies demonstrated the correlation between the risk of MRONJ and the treatment regimen of each antiresorptive agent (18). However, variations in indications and dosages of antiresorptive agents can lead to differences in the risk levels. The risk of MRONJ in patients with bone metastasis who received the oncology doses of IV BPs or Dmab is approximately 1–15%, while the overall risk of MRONJ in osteoporosis patients who received much lower doses of BPs or Dmab is estimated at 0.001–0.01% (34). Furthermore, the route of administration for BPs could play a role in predicting the risk and prognosis of MRONJ, regardless of continuing or discontinuing medication (18, 35). Theoretically, the most commonly used IV BP, zoledronic acid, exhibits greater potency for osteoclast inhibition, higher bone-binding affinity and a longer half-life compared to oral BPs (36). The higher potency, prolonged systemic presence, deep bone penetration and strong inhibition of bone remodeling might potentially serve as contributing factors to advanced-stage MRONJ. This rationale is supported by a histologic jawbone study by Kim *et al.* (37), which demonstrated that patients with MRONJ derived from IV BPs exhibited significantly stronger suppression of osteoclastic marker expression compared to those receiving oral BPs.

Nevertheless, several studies have indicated that discontinuation of both oral and IV BPs may not significantly affect the risk of MRONJ in patients undergoing dental procedures compared to patients who continue drug therapy without interruption. A multicenter study conducted by Kawakita *et al.* (38) and Hasegawa *et al.* (39) demonstrated that a drug holiday during dental extraction in patients who were treated with oral BPs did not reduce the risk of MRONJ when compared to patients who continued medication, together with a study by Karaca *et al.* (30), which was studied in patients receiving IV BPs. The findings from our study strengthen the existing literature by showing that the continuation of oral BPs presents a similar risk of MRONJ compared to patients who discontinued medication before undergoing dental procedures.

When considering IV BPs, which were primarily used in our study to treat bone metastasis, we found that patients had a higher risk of developing MRONJ even if the BPs were discontinued before dental procedures compared to those who continued oral BPs. However, the risk of MRONJ was not different between discontinuation and continuation of IV BPs. Our study suggested that discontinuing both oral and IV BPs for either osteoporosis or bone metastasis before dental procedures may not significantly alter the incidence of MRONJ compared to continuing treatment. This finding could have important implications for clinical practice, as it may indicate that discontinuation of BPs solely for dental procedures might not be necessary in all patients. However, given the limitations in exploring the effects of different durations of drug holidays and the duration of antiresorptive agent use on the risk of MRONJ, it is essential to consider each patient’s specific condition and treatment plan, as well as the potential risks associated with discontinuing BPs therapy.

Dmab drug holiday may cause rebound bone turnover and precipitate an undesirable effect of seriously compromised bone structure (20). The recent treatment guidelines for osteoporosis do not recommend Dmab discontinuation without subsequent therapy with other antiresorptive agents to prevent this rebound phenomenon (3, 4). For bone metastasis patients, a study by Jacobson *et al.* (21) demonstrated that 35% of patients experienced ≥1 SRE following Dmab discontinuation. A retrospective cohort study by Hasegawa *et al.* (40) suggested that both the administration of Dmab and the discontinuation of antiresorptive medication did not result in a significant difference in MRONJ risk among patients receiving antiresorptive medication and undergoing dental procedures. The evidence from our study of Dmab treatment in bone metastasis patients also showed no differentiation in the risk of MRONJ between discontinuing and continuing medication during dental procedures, as well as no significant difference when compared to other types of antiresorptive agents or any discontinuation protocol in terms of MRONJ risk. Furthermore, the risk of MRONJ after Dmab injection for osteoporosis treatment, where the dosage is typically much lower than in malignancy treatment, is generally considered to be very low. These patients should presume tooth extraction if necessary, whereas avoiding tooth extraction can preserve the source of odontogenic infections, which are often associated with the development of MRONJ (41, 42).

Our study has some limitations. The limited number of included studies was insufficient to conduct further subgroup analysis for each indication and type of antiresorptive treatment. In addition, the patients’ characteristics among the included trials lacked uniformity, which could potentially affect the generalizability of the findings. On the contrary, a notable strength of our study is its ability to reflect the diversity and variability of real-world clinical practice. The heterogeneity inherent in the data enhances the relevance of our findings to varied clinical circumstances even though it makes direct comparisons more challenging. By employing a network meta-analysis, we synthesized evidence from diverse treatment modalities, providing a comprehensive overview of their RRs. Furthermore, the patients in this study who received IV BPs and Dmab were predominantly diagnosed with malignancies. This indicates a specific patient population and context that may not directly translate to osteoporosis patients. Healthcare providers need to exercise caution when applying the findings from this study to osteoporosis patients, as the underlying conditions and treatment responses might differ significantly between these patient groups. Nevertheless, given the lower doses and less frequent administration typically used in osteoporosis treatment, the risk of MRONJ in these patients is presumed to be minimal. Finally, the quality of the included studies was mostly rated as having a moderate risk due to within-study bias, which could potentially influence the findings to some extent.

## Conclusion

The risk of MRONJ in patients who discontinued oral BPs and underwent dental procedures is similar to those who continued medication. In addition, temporary discontinuation in patients receiving IV BPs or those on Dmab is unlikely to reduce the risk of MRONJ when compared with continuing medication. This suggests that a drug holiday before dental procedures may not be necessary for patients receiving any antiresorptive agents. This underscores the need for individualized risk assessment and careful management strategies tailored to the specific type of antiresorptive treatment. Further high-quality RCTs are required to strengthen the pooled estimates presented in our study.

## Supplementary materials



## ICMJE Statement of Interest

The authors declare that there is no conflict of interest that could be perceived as prejudicing the impartiality of the work reported.

## Funding Statement

This research received no specific grant from any funding agency in the public, commercial or not-for-profit sectors.
