# Supplementary material for: Effects of discontinuing different antiresorptive regimens on medication-related osteonecrosis of the jaw in patients undergoing dental procedures: a systematic review and network meta-analysis
Source: EFORT Open Rev. 2025 May 5;10(5):258–66. doi: 10.1530/EOR-2024-0133 (PMC12061011; doi:10.1530/EOR-2024-0133)
Supplement: Supplementary file 1 [file supplementary_materials.pdf]

Appendices

Effects of discontinuing different antiresorptive regimens on medication-related osteonecrosis of the jaw in patients undergoing dental procedures: a systematic review and network meta-analysis

Contents

Appendix 1: Searching strategies (from inception to September 2023) 2

1.1 PubMed 2

1.2 EMBASE 3

1.3 CENTRAL 5

1.4 EBSCO Open Dissertation 7

Appendix 2: List of excluded articles and reasons for exclusion 9

Appendix 3: Summary of quality assessment among the included studies 16

Appendix 4: The surface under the cumulative ranking curve (SUCRA) 18

Appendix 5: Assessment of transitivity assumption 19

Appendix 1 Searching strategies (from inception to September 2023)

1.1 PubMed

Search Number	Query	Results
#1	“RANK ligand inhibitor”[Title/Abstract]	48
#2	“RANKL inhibitor” [Title/Abstract]	149
#3	Denosumab[Title/Abstract]	4,259
#4	Prolia[Title/Abstract]	70
#5	Xgeva[Title/Abstract]	38
#6	“AMG 162” [Title/Abstract]	34
#7	Bisphosphonate[Title/Abstract]	11,637
#8	Zoledron*[Title/Abstract]	5,871
#9	Alendron*[Title/Abstract]	5,449
#10	Clodron*[Title/Abstract]	2,467
#11	Etidron*[Title/Abstract]	1,355
#12	Ibandron*[Title/Abstract]	1,171
#13	Pamidron*[Title/Abstract]	2,694
#14	Risedron*[Title/Abstract]	1,939
#15	Antiresorptive[Title/Abstract]	4,179
#16	Bone Density Conservation Agents [MeSH]	16,135
#17	"Denosumab"[Mesh]	2,415
#18	"Diphosphonates"[Mesh]	28,395
#19	"Alendronate"[Mesh]	4,072
#20	"Clodronic Acid"[Mesh]	1,750
#21	"Etidronic Acid"[Mesh]	2,800
#22	"Ibandronic Acid"[Mesh]	776
#23	"Pamidronate"[Mesh]	2,178
#24	"Risedronic Acid"[Mesh]	1,270
**#25**	**OR/1-24**	**45,050**
#26	“tooth extraction” [Title/Abstract]	6,127
#27	"dental extraction"[Title/Abstract]	1,504
#28	“crown lengthening” [Title/Abstract]	546
#29	“Gingivoplasty” [Title/Abstract]	125
#30	“Dental scaling” [Title/Abstract]	256
#31	“Tooth scaling” [Title/Abstract]	25
#32	“Root planing” [Title/Abstract]	3,062
#33	“apicoectomy” [Title/Abstract]	594
#34	“apical surgery” [Title/Abstract]	297
#35	“Osseous surgery” [Title/Abstract]	124
#36	“Endodontic surgery” [Title/Abstract]	504
#37	“Endodontal surgery” [Title/Abstract]	0

Search Number	Query	Results
#38	“Periodontal surgery” [Title/Abstract]	1,722
#39	“Jaw Surgery” [Title/Abstract]	581
#40	“Dental implant” [Title/Abstract]	10,060
#41	Torectomy[Title/Abstract]	4
#42	Alveoloplasty[Title/Abstract]	126
#43	"Crown Lengthening"[Mesh]	554
#44	"Surgery, Oral"[Mesh]	9,363
#45	"Oral Surgical Procedures"[Mesh]	74,763
#46	"Tooth Extraction"[Mesh]	21,448
#47	"Dental Scaling"[Mesh]	4,648
#48	"Root Planing"[Mesh]	2,120
#49	"Apicoectomy"[Mesh]	1,595
#50	"Dental Implants"[Mesh]	29,469
#51	"Dental Implantation"[Mesh]	23,923
#52	"Alveoloplasty"[Mesh]	1,402
#53	"Molar, Third"[Mesh]	7,325
#54	"Orthognathic Surgery"[Mesh]	1,221
#55	"Orthognathic Surgical Procedures"[Mesh]	6,996
**#56**	**OR/26-55**	**114,299**
#57	"Osteonecrosis"[Mesh]	17,688
#58	“Osteonecrosis of the Jaw”	3,090
#59	"Bisphosphonate-Associated Osteonecrosis of the Jaw"[Mesh]	1,715
**#60**	**OR/57-59**	**18,783**
**#61**	**AND/25, 56**	**1,339**
**#62**	**AND/60-61**	**917**

1.2 EMBASE

Search Number	Query	Results
#1	‘rank ligand inhibitor’:ab,ti	83
#2	‘rank inhibitor’:ab,ti	276
#3	denosumab:ab,ti	7,609
#4	prolia:ab,ti	137
#5	xgeva:ab,ti	68
#6	‘amg 162’:ab,ti	37
#7	bisphosphonate:ab,ti	16,488
#8	zoledron*:ab,ti	10,081
#9	alendron*:ab,ti	8,576
#10	clodron*:ab,ti	3,721

Search Number	Query	Results
#11	etidron*:ab,ti	1,728
#12	ibandron*:ab,ti	2,201
#13	pamidron*:ab,ti	4,073
#14	risedron*:ab,ti	3,172
#15	antiresorptive:ab,ti	6,436
#16	'bone density conservation agent'/exp	17,165
#17	‘denosumab’/exp	12,945
#18	‘diphosphonates’/exp	78,204
#19	‘alendronate’/exp	18,703
#20	‘clodronic acid’/exp	7,551
#21	‘etidronic acid’/exp	7,381
#22	‘ibandronic acid’/exp	5,976
#23	‘pamidronate’/exp	11,510
#24	‘risedronic acid’/exp	8,771
**#25**	**OR/1-24**	**89,412**
#26	‘tooth extraction’:ab,ti	6,262
#27	‘dental extraction’:ab,ti	1,947
#28	‘crown lengthening’:ab,ti	507
#29	‘gingivoplasty’:ab,ti	120
#30	‘dental scaling’:ab,ti	132
#31	‘tooth scaling’:ab,ti	27
#32	‘root planing’:ab,ti	2,755
#33	‘apicoectomy’:ab,ti	532
#34	‘apical surgery’:ab,ti	277
#35	‘osseous surgery’:ab,ti	128
#36	‘endodontic surgery’:ab,ti	440
#37	‘endodontal surgery’:ab,ti	0
#38	‘periodontal surgery’:ab,ti	1,582
#39	‘jaw Surgery’:ab,ti	604
#40	‘dental implant’:ab,ti	8,044
#41	torectomy:ab,ti	4
#42	alveoloplasty:ab,ti	116
#43	‘crown lengthening’/exp	253
#44	'oral surgery'/exp	62,826
#45	‘oral surgical procedures’/exp	62,826
#46	‘tooth extraction'/exp	30,518
#47	‘dental scaling'/exp	1,383
#48	‘root planing'/exp	1,166
#49	‘apicoectomy'/exp	311

Search Number	Query	Results
#50	‘dental implants'/exp	20,958
#51	dental implantation'/exp	29,614
#52	‘alveoloplasty'/exp	85
#53	'third molar'/exp	3,354
#54	‘orthognathic surgery'/exp	12,913
#55	‘orthognathic surgical procedures'/exp	12,913
**#56**	**OR/26-55**	**138,283**
#57	‘osteonecrosis'/exp	46,360
#58	'jaw osteonecrosis'/exp	3,985
#59	'bisphosphonate related osteonecrosis of the jaw'/exp	417
**#60**	**OR/57-59**	**46,653**
**#61**	**AND/25, 56**	**2,219**
**#62**	**AND/60-61**	**1,641**

1.3 CENTRAL

Search Number	Searching term	Results
#1	(“RANK ligand inhibitor”):ti,ab,kw	3
#2	(“RANKL inhibitor”):ti,ab,kw	23
#3	(Denosumab):ti,ab,kw	1,286
#4	(Prolia):ti,ab,kw	85
#5	(Xgeva):ti,ab,kw	38
#6	(“AMG 162”):ti,ab,kw	52
#7	(Bisphosphonate):ti,ab,kw	1,581
#8	(Zoledron*):ti,ab,kw	1,818
#9	(Alendron*):ti,ab,kw	1,759
#10	(Clodron*):ti,ab,kw	385
#11	(Etidron*):ti,ab,kw	697
#12	(Ibandron*):ti,ab,kw	491
#13	(Pamidron*):ti,ab,kw	585
#14	(Risedron*):ti,ab,kw	785
#15	(Antiresorptive):ti,ab,kw	549
#16	MeSH descriptor: [Bone Density Conservation Agents] explode all trees	1,955
#17	MeSH descriptor: [Denosumab] explode all trees	460
#18	MeSH descriptor: [Diphosphonates] explode all trees	3,073
#19	MeSH descriptor: [Alendronate] explode all trees	866
#20	MeSH descriptor: [Clodronic Acid] explode all trees	208
#21	MeSH descriptor: [Etidronic Acid] explode all trees	546
#22	MeSH descriptor: [Ibandronic Acid] explode all trees	227

Search Number	Searching term	Results
#23	MeSH descriptor: [Pamidronate] explode all trees	266
#24	MeSH descriptor: [Risedronic Acid] explode all trees	317
**#25**	**OR/1-24**	**7,647**
#26	(“tooth extraction”):ti,ab,kw	3,763
#27	("dental extraction"):ti,ab,kw	296
#28	(“crown lengthening”):ti,ab,kw	78
#29	(“Gingivoplasty”):ti,ab,kw	142
#30	(“Dental scaling”):ti,ab,kw	1,484
#31	(“Tooth scaling”):ti,ab,kw	16
#32	(“Root planing”):ti,ab,kw	2,351
#33	(“apicoectomy”):ti,ab,kw	114
#34	(“apical surgery”):ti,ab,kw	40
#35	(“Osseous surgery”):ti,ab,kw	27
#36	(“Endodontic surgery”):ti,ab,kw	69
#37	(“Endodontal surgery”):ti,ab,kw	0
#38	(“Periodontal surgery”):ti,ab,kw	588
#39	(“Jaw Surgery”):ti,ab,kw	64
#40	(“Dental implant”):ti,ab,kw	1,343
#41	(Torectomy):ti,ab,kw	0
#42	(Alveoloplasty)	60
#43	MeSH descriptor: [Crown Lengthening] explode all trees	16
#44	MeSH descriptor: [Surgery, Oral] explode all trees	260
#45	MeSH descriptor: [Oral Surgical Procedures] explode all trees	5,856
#46	MeSH descriptor: [Tooth Extraction] explode all trees	2,316
#47	MeSH descriptor: [Dental Scaling] explode all trees	1,427
#48	MeSH descriptor: [Root Planing] explode all trees	844
#49	MeSH descriptor: [Apicoectomy] explode all trees	92
#50	MeSH descriptor: [Dental Implants] explode all trees	2,076
#51	MeSH descriptor: [Dental Implantation] explode all trees	1,625
#52	MeSH descriptor: [Alveoloplasty] explode all trees	51
#53	MeSH descriptor: [Molar, Third] explode all trees	1,255
#54	MeSH descriptor: [Orthognathic Surgery] explode all trees	74
#55	MeSH descriptor: [Orthognathic Surgical Procedures] explode all trees	285
**#56**	**OR/26-55**	**12,435**
#57	MeSH descriptor: [Osteonecrosis] explode all trees	350
#58	(Osteonecrosis of the Jaw):ti,ab,kw	251
#59	MeSH descriptor: [Bisphosphonate-Associated Osteonecrosis of the Jaw] explode all trees	39
**#60**	**OR/57-59**	**546**

Search Number	Searching term	Results
**#61**	**AND/25, 56**	**97**
**#62**	**OR/60-61**	**32**

1.4 EBSCO Open Dissertation

Search Number	Searching term	Results
#1	TI “RANK ligand inhibitor” OR AB “RANK ligand inhibitor”	0
#2	TI “RANKL inhibitor” OR AB “RANKL inhibitor”	0
#3	TI Denosumab OR AB Denosumab	0
#4	TI Prolia OR AB Prolia	0
#5	TI Xgeva OR AB Xgeva	0
#6	TI “AMG 162” OR AB “AMG 162”	0
#7	TI Bisphosphonate OR AB Bisphosphonate	82
#8	TI Zoledron* OR AB Zoledron*	15
#9	TI Alendron* OR AB Alendron*	16
#10	TI Clodron* OR AB Clodron*	5
#11	TI Etidron* OR AB Etidron*	3
#12	TI Ibandron* OR AB Ibandron*	0
#13	TI Pamidron* OR AB Pamidron*	2
#14	TI Risedron* OR AB Risedron*	3
#15	TI Antiresorptive OR AB Antiresorptive	1
**#16**	**OR/1-15**	**125**
#17	TI “tooth extraction” OR AB “tooth extraction”	10
#18	TI “dental extraction” OR AB “dental extraction”	1
#19	TI “crown lengthening” OR AB “crown lengthening”	0
#20	TI Gingivoplasty OR AB Gingivoplasty	0
#21	TI “Dental scaling” OR AB “Dental scaling”	0
#22	TI “Tooth scaling” OR AB “Tooth scaling”	0
#23	TI “Root planing” OR AB “Root planing”	4
#24	TI apicoectomy OR AB apicoectomy	0
#25	TI “apical surgery” OR AB “apical surgery”	1
#26	TI “Osseous surgery” OR AB “Osseous surgery”	0
#27	TI “Endodontic surgery” OR AB “Endodontic surgery”	0
#28	TI “Endodontal surgery” OR AB “Endodontal surgery”	0
#29	TI “Periodontal surgery” OR AB “Periodontal surgery”	7
#30	TI “Jaw Surgery” OR AB “Jaw Surgery”	0
#31	TI “Dental implant” OR AB “Dental implant”	65
#32	TI Torectomy OR AB Torectomy	0
#33	TI Alveoloplasty OR AB Alveoloplasty	0

**Search Number**	**Searching term**	**Results**
**#34**	**OR/17-33**	**88**
#35	TI “Osteonecrosis of the Jaw” OR AB “Osteonecrosis of the Jaw”	8
**#36**	**AND/16, 34**	**8**
**#37**	**AND/35-36**	**0**

Appendix 2 List of excluded articles and reasons for exclusion

No.	Authors	Year of publication	Title	Reasons
1.	Aboalela AA et al.	2022	The effect of antiresorptive drug holidays on medication-related osteonecrosis of the jaw: a systematic review and meta-analysis	Systematic review
2.	Aksoy MÇ et al.	2017	Medication related osteonecrosis of the jaws: a case serial study	No intervention of interest
3.	Aljohani S et al.	2018	Osteonecrosis of the jaw in patients treated with denosumab: a multicenter case series	No outcomes of interest
4.	Andriani A et al.	2012	Evolution of bisphosphonate-related osteonecrosis of the jaw in patients with multiple myeloma and Waldenstrom's macroglobulinemia: a retrospective multicentric study	No intervention of interest
5.	Barasch A et al.	2011	Risk factors for osteonecrosis of the jaws: a case-control study from the CONDOR dental PBRN	No outcomes of interest
6.	Boonyapakorn T et al.	2008	Bisphosphonate-induced osteonecrosis of the jaws: prospective study of 80 patients with multiple myeloma and other malignancies	No intervention of interest
7.	Coello-Suanzes JA et al.	2018	Preventive dental management of osteonecrosis of the jaws related to zoledronic acid treatment	No outcomes of interest
8.	Fujieda Y et al.	2020	Incidence and risk of antiresorptive agent□related	No outcomes

			osteonecrosis of the jaw (ARONJ) after tooth extraction in patients with autoimmune disease	of interest
9.	Hadaya D et al.	2021	Antiresorptive-type and discontinuation-timing affect ONJ burden	Animal study
10.	Hamid A et al.	2023	Case series of medication-related osteonecrosis of the jaw (MRONJ) patients prescribed a drug holiday	No intervention of interest
11.	Hasegawa T et al.	2019	Medication-related osteonecrosis of the jaw after tooth extraction in cancer patients: a multicenter retrospective study	No outcomes of interest
12.	Hasegawa T et al.	2013	The observational study of delayed wound healing after tooth extraction in patients receiving oral bisphosphonate therapy	No outcomes of interest
13.	Hinson AM et al.	2015	Temporal correlation between bisphosphonate termination and symptom resolution in osteonecrosis of the jaw: a pooled case report analysis	No outcomes of interest
14.	Kawakita A et al.	2017	Discontinuing oral bisphosphonate therapy during dental extraction does not prevent osteonecrosis of the jaw: a multicenter retrospective study of 341 patients with propensity score matching analysis	No outcomes of interest
15.	Liu FC et al.	2023	Risk comparison of osteonecrosis of the jaw in osteoporotic patients treated	No outcomes of interest

			with bisphosphonates vs. denosumab: a multi-institutional retrospective cohort study in Taiwan	
16.	Schiodt M et al.	2017	Risk of osteonecrosis of the jaws after tooth extraction of 270 teeth with alveolectomy and primary surgical closure in 111 patients on antiresorptive treatment	Abstract proceedings
17.	Ohta R et al.	2015	Clinical review of medication-related osteonecrosis of the jaw regarding risk factors for tooth extraction	Abstract proceedings
18.	Saad F et al.	2012	Incidence, risk factors, and outcomes of osteonecrosis of the jaw: integrated analysis from three blinded active-controlled phase III trials in cancer patients with bone metastases	No intervention of interest
19.	Seki K et al.	2022	Medication-related osteonecrosis of the jaw after tooth extraction in patients receiving pharmaceutical treatment for osteoporosis: a retrospective cohort study	No intervention of interest
20.	Taguchi A et al.	2016	Lack of cooperation between physicians and dentists during osteoporosis treatment may increase fractures and osteonecrosis of the jaw	No experimental study
21.	Taguchi A et al.	2015	Impact of osteonecrosis of the jaw on osteoporosis treatment in Japan: results of a questionnaire-based survey by the adequate treatment of osteoporosis (A-TOP) research	No experimental study

			group	
22.	Thumbigere-Math V et al.	2009	Bisphosphonate-related osteonecrosis of the jaw: clinical features, risk factors, management, and treatment outcomes of 26 patients	No intervention of interest
23.	Villa A et al.	2011	Osteoporosis and bisphosphonate-related osteonecrosis of the jaw bone	No intervention of interest

References

1. Aboalela AA, Farook FF, Alqahtani AS, Almousa MA, Alanazi RT, Almohammadi DS. The effect of antiresorptive drug holidays on medication-related osteonecrosis of the jaw: a systematic review and meta-analysis. *Cureus* 2022; 14(10):e30485. https://doi.org/10.7759/cureus.30485
2. Aksoy MÇ, Koçer G, Koçer M, Baykul T. Medication related osteonecrosis of the jaws: a case serial study. *Int J Clin Exp Med* 2017; 10(11):15672-15679.
3. Aljohani S, Gaudin R, Weiser J, Tröltzsch M, Ehrenfeld M, Kaeppler G, Smeets R, Otto S. Osteonecrosis of the jaw in patients treated with denosumab: A multicenter case series. *J Craniomaxillofac Surg* 2018; 46(9):1515-1525. https://doi.org/10.1016/j.jcms.2018.05.046
4. Andriani A, Petrucci MT, Caravita T, Montanaro M, Villivà N, Levi A, Siniscalchi A, Bongarzoni V, Pisani F, De Muro M, Coppetelli U, Avvisati G, Zullo A, Agrillo A, Gaglioti D. Evolution of bisphosphonate-related osteonecrosis of the jaw in patients with multiple myeloma and Waldenstrom's macroglobulinemia: a retrospective multicentric study. *Blood Cancer J* 2012; 2(3):e62. https://doi.org/10.1038/bcj.2012.9
5. Barasch A, Cunha-Cruz J, Curro FA, Hujoel P, Sung AH, Vena D, Voinea-Griffin AE; CONDOR Collaborative Group; Beadnell S, Craig RG, DeRouen T, Desaranayake A, Gilbert A, Gilbert GH, Goldberg K, Hauley R, Hashimoto M, Holmes J, Latzke B, Leroux B, Lindblad A, Richman J, Safford M, Ship J, Thompson VP, Williams OD, Yin W. Risk factors for osteonecrosis of the jaws: a case-control study from the CONDOR dental PBRN. *J Dent Res* 2011; 90(4):439-444. https://doi.org/10.1177/0022034510397196

6. Boonyapakorn T, Schirmer I, Reichart PA, Sturm I, Massenkeil G. Bisphosphonate-induced osteonecrosis of the jaws: prospective study of 80 patients with multiple myeloma and other malignancies. *Oral Oncol* 2008; 44(9):857-869. https://doi.org/10.1016/j.oraloncology.2007.11.012
7. Coello-Suanzes JA, Rollon-Ugalde V, Castaño-Seiquer A, Lledo-Villar E, Herce-Lopez J, Infante-Cossio P, Rollon-Mayordomo A. Preventive dental management of osteonecrosis of the jaws related to zoledronic acid treatment. *Oral Dis* 2018; 24(6):1029-1036. https://doi.org/10.1111/odi.12842
8. Fujieda Y, Doi M, Asaka T, Ota M, Hisada R, Ohnishi N, Kono M, Kameda H, Nakazawa D, Kato M, Amengual O, Takahata M, Yasuda S, Kitagawa Y, Atsumi T. Incidence and risk of antiresorptive agent-related osteonecrosis of the jaw (ARONJ) after tooth extraction in patients with autoimmune disease. *J Bone Miner Metab* 2020; 38(4):581-588. https://doi.org/10.1007/s00774-020-01089-y
9. Hadaya D, Soundia A, Gkouveris I, Bezouglaia O, Dry SM, Pirih FQ, Aghaloo TL, Tetradis S. Antiresorptive-Type and Discontinuation-Timing Affect ONJ Burden. *J Dent Res* 2021; 100(7):746-753. https://doi.org/10.1177/0022034520986804
10. Hamid A, Thomas S, Bell C, Gormley M. Case series of medication-related osteonecrosis of the jaw (MRONJ) patients prescribed a drug holiday. *Br J Oral Maxillofac Surg* 2023; 61(3):227-232. https://doi.org/10.1016/j.bjoms.2023.02.003
11. Hasegawa T, Hayashida S, Kondo E, Takeda Y, Miyamoto H, Kawaoka Y, Ueda N, Iwata E, Nakahara H, Kobayashi M, Soutome S, Yamada SI, Tojyo I, Kojima Y, Umeda M, Fujita S, Kurita H, Shibuya Y, Kirita T, Komori T. Medication-related osteonecrosis of the jaw after tooth extraction in cancer patients: a multicenter retrospective study. *Osteoporos Int* 2019; 30(1):231-239. https://doi.org/10.1007/s00198-018-4746-8
12. Hasegawa T, Ri S, Umeda M, Komatsubara H, Kobayashi M, Shigeta T, Yoshitomi I, Ikeda H, Shibuya Y, Asahina I, Komori T. The observational study of delayed wound healing after tooth extraction in patients receiving oral bisphosphonate therapy. *J Craniomaxillofac Surg* 2013; 41(7):558-563. https://doi.org/10.1016/j.jcms.2012.11.023
13. Hinson AM, Siegel ER, Stack BC Jr. Temporal correlation between bisphosphonate termination and symptom resolution in osteonecrosis of the jaw: a pooled case report analysis. *J Oral Maxillofac Surg* 2015; 73(1):53-62. https://doi.org/10.1016/j.joms.2014.07.012

14. Kawakita A, Yanamoto S, Morishita K, Naruse T, Hayashida S, Soutome S, Rokutanda S, Inokuchi S, Matsuo T, Umeda M. Discontinuing oral bisphosphonate therapy during dental extraction does not prevent osteonecrosis of the jaw: a multicenter retrospective study of 341 patients with propensity score matching analysis. *J Oral Maxillofac Surg Med Pathol* 2017; 29(6):522-526. https://doi.org/10.1016/j.ajoms.2017.07.008
15. Liu FC, Luk KC, Chen YC. Risk comparison of osteonecrosis of the jaw in osteoporotic patients treated with bisphosphonates vs. denosumab: a multi-institutional retrospective cohort study in Taiwan. *Osteoporos Int* 2023; 34(10):1729-1737. https://doi.org/10.1007/s00198-023-06818-3
16. Schiodt M, Ottesen C, Madsen S, Nielsen E, Sand L, Gjoedesen C. Risk of osteonecrosis of the jaws after tooth extraction of 270 teeth with alveolectomy and primary surgical closure in 111 patients on antiresorptive treatment. *Int J Oral Maxillofac Surg* 2017; 46:113. https://doi.org/10.1016/j.ijom.2017.02.399
17. Ohta R, Onda T, Morikawa T, Ogane S, Nomura T, Takano N, Shibahara T. Clinical review of medication-related osteonecrosis of the jaw regarding risk factors for tooth extraction. *Int J Oral Maxillofac Surg* 2015; 44:e270. https://doi.org/10.1016/j.ijom.2015.08.265
18. Saad F, Brown JE, Van Poznak C, Ibrahim T, Stemmer SM, Stopeck AT, Diel IJ, Takahashi S, Shore N, Henry DH, Barrios CH, Facon T, Senecal F, Fizazi K, Zhou L, Daniels A, Carrière P, Dansey R. Incidence, risk factors, and outcomes of osteonecrosis of the jaw: integrated analysis from three blinded active-controlled phase III trials in cancer patients with bone metastases. *Ann Oncol* 2012; 23(5):1341-1347. https://doi.org/10.1093/annonc/mdr435
19. Seki K, Kaneko T, Kamimoto A, Wada M, Takeuchi Y, Furuchi M, Iinuma T. Medication-related osteonecrosis of the jaw after tooth extraction in patients receiving pharmaceutical treatment for osteoporosis: A retrospective cohort study. *J Dent Sci* 2022; 17(4):1619-1625. https://doi.org/10.1016/j.jds.2022.03.014
20. Taguchi A, Shiraki M, Sugimoto T, Ohta H, Soen S. Lack of cooperation between physicians and dentists during osteoporosis treatment may increase fractures and osteonecrosis of the jaw. *Curr Med Res Opin* 2016;32(7): 1261-1268. https://doi.org/10.1185/03007995.2016.1170005
21. Taguchi A, Shiraki M, Tsukiyama M, Miyazaki T, Soen S, Ohta H, Nakamura T, Orimo H. Impact of Osteonecrosis of the Jaw on Osteoporosis Treatment in Japan: Results of a

Questionnaire-Based Survey by the Adequate Treatment of Osteoporosis (A-TOP) Research Group. *Calcif Tissue Int* 2015;97(6): 542-550. https://doi.org/10.1007/s00223-015-0045-y

22. Thumbigere-Math V, Sabino MC, Gopalakrishnan R, Huckabay S, Dudek AZ, Basu S, Hughes PJ, Michalowicz BS, Leach JW, Swenson KK, Swift JQ, Adkinson C, Basi DL. Bisphosphonate-related osteonecrosis of the jaw: clinical features, risk factors, management, and treatment outcomes of 26 patients. *J Oral Maxillofac Surg* 2009; 67(9):1904-1913. https://doi.org/10.1016/j.joms.2009.04.051
23. Villa A, Castiglioni S, Peretti A, Omodei M, Ferrieri GB, Abati S. Osteoporosis and bisphosphonate-related osteonecrosis of the jaw bone. *ISRN Rheumatol* 2011; 2011:654027. https://doi.org/10.5402/2011/654027

Appendix 3 Summary of quality assessment among the included studies

3.1 The risk of bias in non-randomized studies of interventions (ROBINS-I)

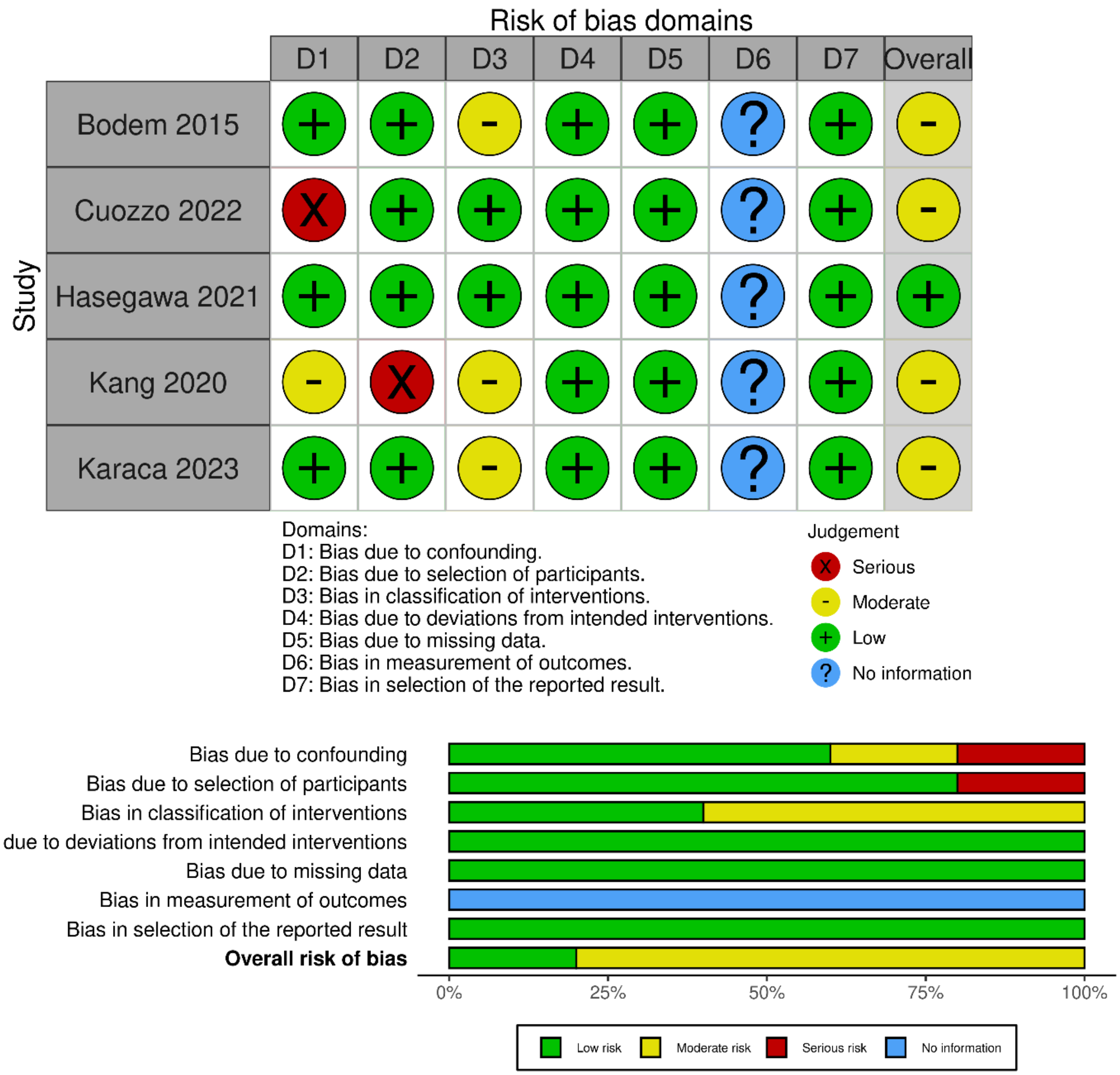

Appendix Figure 1 Quality evaluation according to the ROBINS-I tool for the non-randomized studies.

3.2 The risk of bias in randomized trials (RoB 2)

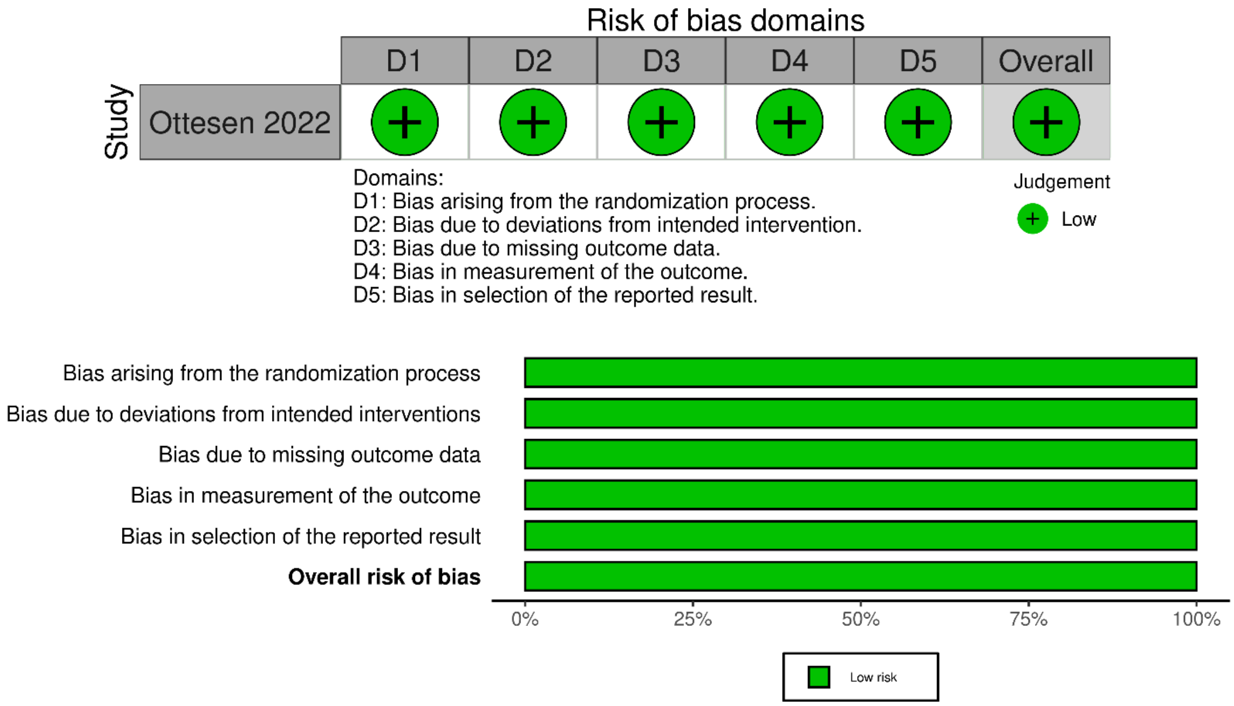

Appendix Figure 2 Quality evaluation according to the RoB2 tool for the RCTs.

Appendix 4 The surface under the cumulative ranking curve (SUCRA)

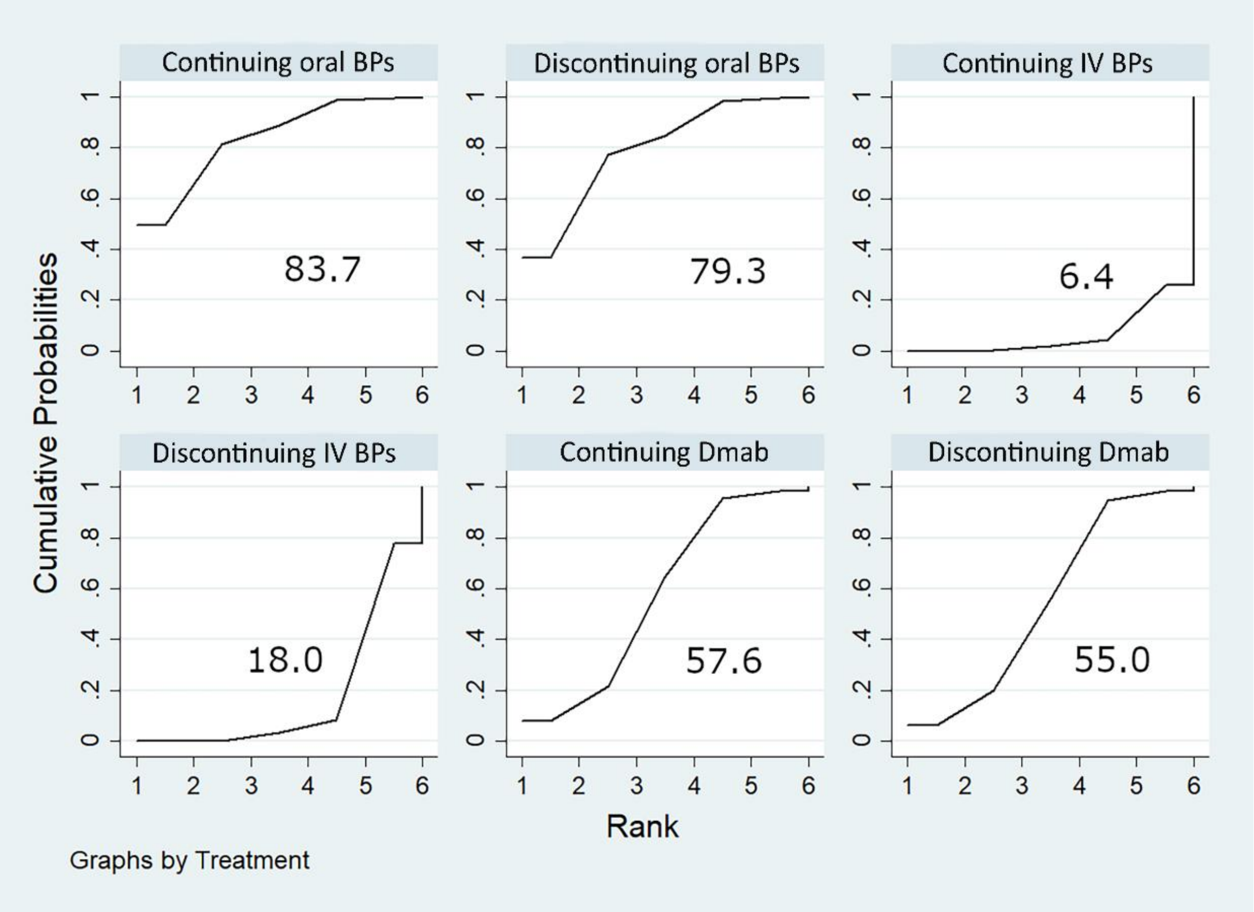

Appendix Figure 3 The graph shows SUCRA ranking related to the efficacy of different modalities.

Appendix 5 Assessment of transitivity assumption

Treatment comparison	No. of study	No. of subject	Mean age (years)	Female (%)	Duration of antiresorptive treatment (months)	Duration of antiresorptive discontinuation (months)	Duration of follow-up after dental procedures (months)
Discontinuing oral BPs vs Continuing oral BPs	2	454	67.5	95.6	N/A	3	12
Continuing IV BPs vs Discontinuing oral BPs	1	275	N/A	N/A	N/A	N/A	N/A
Continuing IV BPs vs Discontinuing IV BPs	4	148	57.2 – 65.7	50.0 – 72.5	3 – 245	0 – 96	2 – 6
Discontinuing IV BPs vs Continuing Dmab	1	10	N/A	50.0 – 53.8	2 – 96	0 – 4	6
Continuing Dmab vs Discontinuing Dmab	2	85	65.2	53.8 – 56.9	1 – 85	0 – 4	6 – 24
Continuing IV BPs vs Continuing Dmab	1	10	N/A	50.0 – 53.8	2 – 96	0	6
Continuing IV BPs vs Discontinuing Dmab	1	13	N/A	50.0 – 53.8	2 – 96	0 – 4	6
Continuing IV BPs vs Continuing oral BPs	1	164	N/A	N/A	N/A	0	N/A
Discontinuing IV BPs vs Discontinuing oral BPs	1	271	N/A	N/A	N/A	N/A	N/A
Discontinuing IV BPs vs Continuing oral BPs	1	160	N/A	N/A	N/A	N/A	N/A
Discontinuing IV BPs vs Discontinuing Dmab	1	13	N/A	50.0 – 53.8	2 – 96	4	6

Abbreviation: IV, intravenous; BPs, bisphosphonates; Dmab, denosumab; N/A, not applicable
